# Psychological Neuromodulatory Treatments for Young People with Chronic Pain

**DOI:** 10.3390/children3040041

**Published:** 2016-12-06

**Authors:** Jordi Miró, Elena Castarlenas, Rocío de la Vega, Rubén Roy, Ester Solé, Catarina Tomé-Pires, Mark P. Jensen

**Affiliations:** 1 Chair in Pediatric Pain URV-Fundación Grünenthal, Unit for the Study and Treatment of Pain, ALGOS, 43007 Tarragona, Catalonia, Spain; elenateresa.castarlenas@urv.cat (E.C.); rocio.delavega@urv.cat (R.d.l.V.); ruben.roy@urv.cat (R.R.); ester.sole@urv.cat (E.S.); catarina.tome-pires@urv.cat (C.T.-P.); 2 Research Center for Behavior Assessment (CRAMC), Department of Psychology, 43007 Tarragona, Catalonia, Spain; 3 Institut d’Investigació Sanitària Pere Virgili, Reus 43202, Catalonia, Spain; 4 Department of Rehabilitation Medicine, University of Washington, Seattle, WA 98105, USA; mjensen@uw.edu

**Keywords:** pediatric chronic pain, psychological neuromodulatory treatments, hypnosis, meditation, mindfulness, neurofeedback, efficacy

## Abstract

The treatment of young people with chronic pain is a complex endeavor. Many of these youth do not obtain adequate relief from available interventions. Psychological neuromodulatory treatments have been shown to have potential benefit for adults with chronic pain. Here, we review and summarize the available information about the efficacy of three promising psychological neuromodulatory treatments—neurofeedback, meditation and hypnosis—when provided to young people with chronic pain. A total of 16 articles were identified and reviewed. The findings from these studies show that hypnotic treatments are effective in reducing pain intensity for a variety of pediatric chronic pain problems, although research suggests variability in outcomes as a function of the specific pain problem treated. There are too few studies evaluating the efficacy of neurofeedback or meditation training in young people with chronic pain to draw firm conclusions regarding their efficacy. However, preliminary data indicate that these treatments could potentially have positive effects on a variety of outcomes (e.g., pain intensity, frequency of pain episodes, physical and psychological function), at least in the short term. Clinical trials are needed to evaluate the effects of neurofeedback and meditation training, and research is needed to identify the moderators of treatment benefits as well as better understand the mechanisms underlying the efficacy of all three of these treatments. The findings from such research could enhance overall treatment efficacy by: (1) providing an empirical basis for better patient-treatment matching; and (2) identifying specific mechanisms that could be targeted with treatment.

## 1. Introduction

Pediatric chronic pain is a common problem worldwide. Although the reported prevalence rates vary as a function of how chronic pain is defined and the reporting period used, the available evidence indicates that pediatric chronic pain is a serious public health problem: prevalence rates range from 6% to 37% [1,2,3]. The most common pediatric chronic pain problems are headaches, abdominal pain and musculoskeletal pain.

Chronic pain has been defined as “pain without apparent biological value that has persisted beyond normal tissue healing time” [3]. It is a complex experience resulting from the interaction of biological, cognitive, emotional, behavioral, and cultural factors. Chronic pain is known to have significant negative effects on the lives of children and adolescents. For example, research has shown that young people with chronic pain have more cognitive and emotional problems and pain-related worries than their peers without pain [4,5]. Young people with chronic pain also experience more sleep problems [6,7], disability [8,9], impaired social relations [10,11], problems in school function [12,13] and, in general, poorer perceived quality of life [14,15] than their otherwise healthy peers. Moreover, the negative effects of chronic pain also extend to others who live with these youth. For example, family members of children with chronic pain experience more emotional distress, limitations in daily functioning, and problems in marital and social relationships [16,17] than family members of children without chronic pain. Chronic pain also has a significant financial cost, including both direct (e.g., cost of analgesics) and indirect costs (e.g., loss of productivity because one of the parents must stay at home to care for the youth with chronic pain) [18].

Biomedical treatments, consisting primarily of analgesics, are the most common therapeutic strategy used to manage pediatric chronic pain. However, even with the most sophisticated biomedical interventions, many youths continue to experience significant chronic pain and its negative effects [19]. In fact, many of the compounds used in the treatment of pediatric populations with chronic pain have not been licensed for that particular purpose or for the specific pain indication for which they are used with youth [20].

Non-pharmacological treatment options are also sometimes offered to children with chronic pain, although such treatments have traditionally been used as a secondary option (i.e., secondary to the biomedical approach) or as a “last resort” when biomedical treatments are not found to be effective [21]. However, the understanding that chronic pain results from the interaction of biological, emotional, cognitive, behavioral and social factors has led to the promotion of biopsychosocial interventions that address each of these factors [20,22,23,24]. Furthermore, with the conceptualization of pain as a multidimensional and biopsychosocial phenomenon, and the recognition that pain is ultimately created by the brain (i.e., it is the result of the cortical processing of sensory information in combination with an individual’s learning history and evaluations of those sensations), there has been an increased interest in interventions that target brain activity directly to reduce pain and pain-related suffering—so-called psychological neuromodulatory interventions.

There are a number of psychological neuromodulatory treatments that have demonstrated efficacy in adults with chronic pain, and there is a growing number of clinical trials evaluating their efficacy in pediatric populations [25]. The objective of this review paper is to summarize the state of knowledge about the efficacy of three of these psychological neuromodulatory treatments when used with youth with chronic pain: neurofeedback, meditation, and hypnosis. In the sections that follow, and for each of these treatments, we provide a definition and description of the treatment, and then a review of the available evidence regarding its efficacy in youth with chronic pain. The paper ends with a discussion of the clinical implications of the findings and potential avenues for future research.

In order to identify the research studies in this area, we searched the PsycINFO, MEDLINE, PsycARTICLES, Health and Psychosocial Instruments, CINAHL, Scopus and ProQuest Dissertations and Theses databases from their inception to July 2016, using the search terms “((neurofeedback OR meditation OR hypnosis) AND chronic pain AND (child* OR adolescent OR infant OR pediatric OR paediatric OR young OR youth) AND (treatment OR management))”. We focused on empirical articles that dealt with chronic non-cancer pain. We also identified systematic reviews which would help to provide the most updated and comprehensive information possible on the topic, both through the original electronic search and through the reference lists of the identified articles. Studies with participants up to 21 years of age are included in this review.

## 2. Neurofeedback

### 2.1. Definition and Procedures

Neurofeedback (NF) is a type of biofeedback treatment that provides real-time information to the patient about his or her brain activity, allowing patients to learn how to directly change this activity in ways that are thought to be healthier, more efficient, or provide comfort. As stated by the International Society for Neurofeedback and Research [26], what distinguishes NF from other forms of biofeedback is its focus on the central nervous system; in particular the brain. The assessment of brain activity is performed either through Electroencephalography (EEG) or functional Magnetic Resonance Imaging (fMRI) [25]. The EEG alternative is used much more often because of practical and economic reasons [25]. No studies have examined the efficacy of fMRI-based biofeedback in youth with chronic pain. Therefore, in this review, all references to NF are references to EEG biofeedback.

Although there are a variety of NF protocols, they share the same basic procedures, which are based on operant learning principles [27]. All NF procedures use a Brain Computer Interface; that is, a communication system between the brain and some external device [28]. Briefly, with NF, one or more electrodes is/are placed on the patient’s scalp to assess his or her electrical brain activity. The raw electrical signal (which represents the collective activity of hundreds of thousands of neurons in the cortex just below the electrode) is digitalized and the amplitudes of activity in specific frequency bandwidths (e.g., alpha activity, in the 8–12 Hz range) are displayed on the therapist’s monitor. Depending on the bandwidths that are the target of treatment—usually bandwidths thought to represent dysfunctional activity—a specific treatment protocol is developed. This protocol is designed to enhance (or inhibit, as appropriate) the power or amount of specific oscillations in order to achieve the therapeutic goals (e.g., increase alpha activity to facilitate perceived relaxation, increase beta or gamma bandwidth activity over the motor cortex to facilitate the inhibition of sensory signals, increase theta or alpha activity over the sensory cortex to inhibit the processing of sensory information, etc.).

Activity in the targeted bandwidth is then “fed back” to the patient, along with instructions to increase or decrease that activity. In children, the feedback is often presented as a game. For example, they are instructed to “fly the rocket” and the software is written to provide the patient with an image of a flying rocket if and when the brain activity changes or maintains in the direction of the training criteria established by the therapist. This feedback enables the patient to influence and progressively change brainwave patterns via operant conditioning [27,29,30]. There are a variety of treatment protocols that are usually named based on the frequencies they intend to alter (e.g., a “Beta protocol” would be one aimed at modifying beta oscillations). It can take a relatively long time for brain activity changes to occur with NF treatment; a full course of NF treatment is normally comprised by 15 to 50 sessions of 20–40 min each [29,30,31].

Individuals with chronic pain show patterns of EEG activity that differ from those without chronic pain. For example, Pinheiro and colleagues [32] found that individuals with chronic pain display increased alpha and theta frequencies at rest, relative to those without chronic pain [32]. NF treatment for pain usually targets these and other bandwidths in order to: (1) decrease the processing of sensory information; (2) increase activity in areas of the brain that operate to inhibit sensory information; and/or (3) increase perceived relaxation [25]. The electrode placement sites used in the treatment of pain vary, and depend on the specific brain activity patterns thought to underlie a patient’s unique pain problem. Electrode placement sites often include central and temporal areas of the cortex [33], but can include as many as 19 different sites in the course of a patient’s treatment program [34].

The typical NF session begins with a 2–3 min resting condition to assess baseline EEG activity, which is then followed by several training trials spaced by small breaks. During the training trials, the parameters are set such that one (or more) oscillation bandwidth is reinforced and/or one (or more) oscillation bandwidth is “inhibited” (i.e., the patient needs to demonstrate a decrease in activity in that bandwidth in order to receive the reinforcement). For example, in one recent study evaluating the efficacy of NF to treat chronic neuropathic pain in adults, the investigators reinforced alpha frequency bandwidth (8–12 Hz) activity, and inhibited theta (4–8 Hz) and higher beta (20–30 Hz) bandwidth activity [33]. Reward thresholds are progressively adjusted so that the visual and/or auditory feedback reward is provided to the patient 60%–70% of the time [29].

### 2.2. Effects

To the best of our knowledge, only two studies have reported on the effects of NF when used for the treatment of youth with chronic pain. Although there are a number of studies that have included adolescents in their samples (e.g., [35,36]), the way in which the data are reported in these studies does not allow us to evaluate the results separately for the children versus adults. Therefore, they were not included in this review.

Siniatchkin and colleagues [37] examined the efficacy of Slow Cortical Potentials NF in a very small sample (*n* = 10) randomized controlled trial of children with migraine without aura. Participants in this trial (which included two control groups; see Table 1) were provided visual feedback in the form of a bar. The protocol was established so that the bar became longer with brain activity thought associated with increased autonomic muscular tension, and shorter with brain activity thought associated with increased relaxation (i.e., soothing thoughts and a restful state). The participants in this study were also asked to pay attention to the thoughts or body sensations that helped them to perform the task. After 10 sessions provided over an eight-week period, the treatment group showed significant reductions in the number of days with migraine per month and the duration of migraine episodes; effects that were not found in the control groups. However, no statistically significant improvements were found in the treatment group on measures assessing the intensity of the migraine headaches, the use of headache medications or other migraine-related symptoms.

In a case series (uncontrolled) study, Stokes and Lappin [38] treated 37 patients with migraine with a combination of NF, passive infrared hemo-encephalography (pIR-HEG), and thermal biofeedback. Thirteen of the study participants were up to 21 years of age, and here we report the results from this subset of patients. The treatment consisted of an average of 40 sessions and included an average of 30 frequency-based NF sessions and an average of 10 pIR-HEG or hand-warming biofeedback session provided over the course of six months. NF training aimed to reduce the amplitude of the frequencies which were assessed at baseline and determined to be “excessive”; that is, treatment was tailored to each participant and was not standardized. The use of a combination of treatments as well as the lack of a control condition does not allow us to determine how much of this benefit was due to the specific effects of NF treatment, how much was due to the specific effects of the other treatment components, and how much was due to non-specific effects (e.g., time, outcome expectancies, therapist attention, etc.).

### 2.3. Conclusions

NF is an intervention still under development and evaluation for chronic pain. Despite some preliminary encouraging results in adults [25], the lack of controlled trials does not allow us to conclude that NF is an effective treatment for chronic pain in adolescents and children with chronic pain. Nevertheless, the two studies identified do provide preliminary evidence indicating the possibility that NF may benefit young people with chronic pain. Controlled trials are needed to evaluate this possibility. If found to be effective, research is also needed to: (1) understand which NF treatment protocols (number of sessions, electrode training sites, oscillations to reinforce and inhibit, necessity of “booster” treatment sessions, etc.) produce the best results; (2) understand the mechanisms that explain treatment benefits; and (3) identify the patient factors that predict positive treatment responses.

## 3. Meditation

### 3.1. Definition and Procedures

Meditation has been defined as “a family of complex emotional and attentional regulatory strategies developed for various ends, including the cultivation of well-being and emotional balance” [39]. Traditionally, meditation procedures have been classified into two types: focused attention meditation and open monitoring meditation [39]. Focused attention meditation includes focusing and sustaining the attention on a specific object (e.g., a flame) or an internally generated image or sound (e.g., image of a flame or mantra), noting when attention begins to wander from that object, and then refocusing attention back onto the object. Open monitoring meditation promotes focusing on and being aware of and accepting without judgment all elements of one’s “present experience” without purposefully focusing on any one image or object.

Mindfulness, which is a type of monitoring meditation, is the strategy that has been studied the most in relation to the management of chronic pain in both youths and adults [25]. This procedure has been defined by Kabat-Zinn [40] as “the awareness that emerges through paying attention on purpose, in the present moment, and non-judgmentally to the unfolding of experience, moment by moment”. Three common standardized interventions that include mindfulness practice are: (1) Mindfulness–Based Stress Reduction (MBSR) [41]; (2) Mindfulness-based Cognitive Therapy (MBCT) [42]; and (3) Acceptance and Commitment Therapy (ACT) [43].

Mindfulness-based interventions have been described as effective approaches to manage chronic health conditions in young people [44,45], such as in cancer [46] or depression [47]. There are fewer studies evaluating the efficacy of mindfulness in pediatric populations with chronic pain than in adult populations [48,49], although the number of studies using youth samples is growing. In the section that follows, we focus in the results from studies of mindfulness related interventions when used to manage chronic pain in pediatric populations.

### 3.2. Effects

Mindfulness approaches have been recently used with adolescents with chronic pain, either by using an adaptation of the MBSR program that was original developed for adults [50,51,52] or as a part of ACT treatment (although mindfulness training is just one of several treatment components of ACT) [53,54]. These interventions have all been implemented in a group format. The available studies have mostly sought to examine the feasibility and acceptability of these treatments; preliminary efficacy data are only reported in a few cases.

It is difficult to compare the results of the available studies due to methodological differences, such as the type of study design (case series versus randomized clinical trial), the content of the mindfulness treatment component, the inclusion of non-mindfulness-based treatment components, the number of treatment sessions provided, and the amount of time of recommended home meditation practice (see Table 1 for a detailed description of the studies reviewed here). Generally speaking, these interventions that include training in mindfulness meditation are well accepted by adolescents with chronic pain, as reflected by the high attendance rates to sessions and high compliance with home practice recommendations. For example, 81% of the participants in one study [52] and 75% in another [50] completed the interventions.

Preliminary results suggest that the MBSR program has significant potential for efficacy (see Table 1). To the best of our knowledge, the first randomized controlled trial using MBSR with pediatric populations with chronic pain was conducted by Jastrowski and colleagues [51]. This study included a very small sample of participants, with four children in a MBSR condition and two in a psychoeducation group. They found that all six participants, regardless of treatment condition, reported increases in mindfulness self-efficacy, but they found inconsistent results regarding the other outcome variables. For example, only three of the participants (two in the mindfulness condition and one in the psychoeducation condition) reported improvements on the total score of a measure assessing physical, emotional, social, and school function.

Two additional studies have adapted the MBSR to be used with adolescents. In one of these studies, Hesse and colleagues [50] conducted a case series study with 20 adolescents with recurrent headache. After eight weeks of treatment, the participants reported a reduction in depressive symptoms and an increase in “pain willingness”; that is, an increase in the belief that pain control is not as important as other life goals. However, no significant pre- to post-treatment differences were found in the other outcome domains, including disability, or anxiety or physical, emotional, social, and school function.

Ruskin and colleagues [52] also adapted MBRS treatment for adolescents with chronic pain and implemented it in a sample of 16 adolescents with a variety of chronic pain conditions (e.g., musculoskeletal pain, abdominal pain). At the end of the treatment, the study participants reported that the treatment helped them to cope better with their pain and to manage their emotions. These results were based on qualitative interviews with the small number of participants who completed the whole program and the assessment at follow-up (see Table 1 for additional details).

Mindfulness techniques have also been used with adolescents with chronic pain as a component of an ACT program [53,54]. Gauntlett-Gilbert and colleagues [53] used a case series study to evaluate changes that occurred following an ACT residential pain management program in a sample of 98 adolescents with chronic pain. The results showed pre- to post-treatment improvements in most of the outcome variables, such as, physical and social disability, pain-related anxiety or pain acceptance, which were maintained for the most part, at the three-month follow-up (see Table 1 for detailed information).

In a more recent study, Martin and colleagues [54] examined changes in outcomes following three two-hour sessions over two days of ACT treatment, which included a number of mindful procedures such as mindful breathing, in a sample of 10 adolescents with neurofibromatosis type 1 and chronic pain. After completion of treatment, the study participants reported significant reductions in pain intensity and pain interference, which maintained at the three-month follow-up assessment point. However, no significant pre- to post-treatment improvements were observed in other outcomes such as pain acceptance, anxiety, and depression.

### 3.3. Conclusions

Taken as a whole, the findings from research on meditation approaches when used with young people with chronic pain support their feasibility and acceptability. However, data regarding the efficacy of this treatment approach are almost non-existent. The preliminary uncontrolled findings suggest the possibility that mindfulness may help adolescents with chronic pain to improve some (but not all) quality of life domains, including functional status. However, there remain significant methodological limitations that should be addressed in future research. One noteworthy limitation, common for most of the studies, is the small sample sizes. Another related limitation is the quality of the study design. Only one randomized controlled trial has been published [51], but this study was conducted with an extremely small sample, severely limiting the conclusions that can be drawn from the findings. In addition, in many of the studies performed to date, mindfulness training is just one component of a multi-component treatment “package” (e.g., ACT). While it might be argued that the MBSR is at its core a mindfulness training program, we were only able to identify three studies that examined the effects of MBSR (see Table 1). Two of these were case series studies and one was a randomized trial with very few (*n* = 6) subjects. Thus, there is a need for more studies to examine the specific effects of training in mindfulness practice. Furthermore, studies using other meditation procedures, like focused attention meditation, are also needed.

Moreover, the field still needs to reach a consensus regarding how best to adapt mindfulness-based interventions for young people with chronic pain. For example, it would be important to establish the number of sessions needed to maximize any benefits, as well as the number of home-practice hours needed in order to assimilate and integrate the meditation practice into the lives of these youth. In addition, research is also needed to identify the person variables, both internal and external personal characteristics, that facilitate the acquisition of knowledge and skills required in meditation practice. Furthermore, the current research is also limited by the use of participants who are 10 years old or older; future research is needed to evaluate the feasibility and efficacy of meditation practices in children younger than 10 years old.

## 4. Hypnosis

### 4.1. Definition and Procedures

Division 30 of the American Psychological Association (APA) defines hypnosis as a “State of consciousness involving focused attention and reduced peripheral awareness characterized by an enhanced capacity for response to suggestion” [55]. Hypnotic procedures usually start with a hypnotic “induction” (thought to increase the patient’s response to suggestions), followed by specific suggestions that target the presenting problem. A hypnotic induction can include a number of strategies (e.g., suggestions for relaxation, counting methods) [56,57]. In pediatric chronic pain it is common to use relaxation and/or imagery as induction techniques [58].

In the context of pain management, a typical hypnotic protocol can include suggestions for greater comfort or for coping more adequately with pain, ranging, for example, from direct instructions (e.g., “*You are noticing where in the body you feel the greatest comfort, and can allow this sense of comfort to expand…*”) to metaphors (e.g., “*You might visualize any uncomfortable sensations as an image or object, such as a fire or tightly knotted rope,... and now notice how that object changes and as you notice these changes, your experience changes... becoming more and more comfortable*”), to general comfort images (e.g., “*…floating like a big comfortable cloud*”) or specific suggestions for the problem that is being addressed (e.g., for abdominal pain it can be suggested to patients to “*Feel your hands as warm and place both hands on your abdomen, imagining the warmth spreading into and throughout the abdomen…*”) [25,58,59]. Treatment also often includes post-hypnotic suggestions given at the end of the hypnotic sessions which are designed to maintain and extend the therapeutic benefits achieved during the sessions into the patient’s daily life. Often, patients are given an audio recording of the sessions to facilitate home practice [58,60].

### 4.2. Effects

Previous reviews summarizing the findings from randomized controlled trials (RCTs) [58,59], and case studies and non-controlled studies [61], have shown the positive effects of hypnosis when used in the treatment of youth with chronic pain. Most studies for chronic non-cancer related pain have been conducted in samples if children with headache or functional abdominal pain. The results are generally positive, either as presented in uncontrolled case series studies using hypnosis only [62,63,64] or in combination with other treatments such as acupuncture [65]. The most consistent beneficial effects are found for pain intensity [66,67,68] and pain frequency [67,68,69] in samples of children with abdominal pain. These benefits maintain at up to five-year follow-up (see a description of reviewed studies in Table 1) [68].

However, the findings are not as consistently positive in samples of young people with headache. For example, Olness and colleagues [70] found that a hypnotic protocol consisting of standard progressive relaxation exercise, imagery and hypnotic suggestions (e.g., hand anesthesia) significantly reduced the number of headaches but did not decrease headache pain intensity. Positive effects in controlled studies have been also found in samples of children with abdominal pain, in the reduction of disability [66] and school absenteeism [69], although not all studies have reported significant effects on absenteeism [66,67,68].

### 4.3. Conclusions

On the basis of the available research, summarized here, it appears that the use of hypnosis for the management of pediatric chronic pain is promising, although its efficacy also appears to vary as a function of the pain condition studied. For example, the efficacy of hypnosis as a treatment for youth with chronic abdominal pain has strong research support whereas its use in the management of headaches only has modest support (based on the criteria proposed by the Society of Clinical Psychology, Division 12 of the American Psychological Association [71], recently updated, see https://www.div12.org/faq/). There are a variety of hypnotic strategies available which can target a variety of outcomes and mechanisms. However, there is not yet any evidence regarding which protocols are most effective for whom and under what circumstances. There is a general belief, however, that treatment should be tailored to the age of the patient for best results [72].

Future research is needed to identify which hypnosis treatment protocols are most effective, and if the efficacy of protocols are moderated by type of pain and age of the patient or other factors. In addition, many investigators and clinicians have expressed the belief that hypnotic suggestions should target the multidimensional experience and effects of pain, including pain intensity, psychological distress (e.g., anxiety), maladaptive thoughts (e.g., catastrophizing), as well as other domains known to be influenced by pain, such as analgesic intake and sleep quality [73,74,75]. Research is needed to evaluate this assumption, and to identify which outcome domains are most responsive to hypnotic treatment.

## 5. Conclusions and General Discussion

The results from this review indicate that psychological neuromodulatory treatments are promising treatments for young people with chronic pain. However, there are more findings supporting the efficacy of hypnosis—in particular for chronic abdominal pain—than for either neurofeedback or meditation. In this review, we limited our focus to non-cancer chronic pain-related problems. However, the conclusions from this review—at least those conclusions with respect to the efficacy of hypnosis—are consistent with those from reviews of studies in youth with cancer-related pain (e.g., [58,76]), providing even more support for the efficacy of hypnosis. An additional finding supporting the use of hypnosis for chronic pain in youth—even as a potential “first line treatment”—is its side effect profile; that is, the benefits noted appear to occur in the absence of any significant negative side effects, other than perhaps the effort and time it takes to learn the self-hypnosis skills taught [58].

Not all patients benefit from hypnotic treatment, however. For example, the rates of children who benefited from hypnosis as reported in the studies reviewed here ranged from 67% to 96% (see Table 1). Moreover, we still do not understand the factors that predict outcome. We still do not know for whom hypnotic treatment works best, and which of the many possible hypnotic protocols produce the most benefit. There is a belief among those who are not familiar with hypnosis research that trait hypnotizability predicts response to hypnosis treatment. However, while the association between measures of hypnotizability and hypnosis treatment in adults tends to be positive, the strength of those associations also tends to be weak [77]; high levels of trait hypnotizability do not appear to be necessary to obtain benefits from hypnotic treatments. On the other hand, there is some evidence that adult patients with neuropathic pain may respond better to hypnosis treatment than patients with non-neuropathic chronic pain conditions [25]. However, the generalizability of this finding to children is not known. In short, even as the field continues to evaluate the efficacy of hypnosis for chronic pain conditions in youth, there is also a need to better understand the mechanisms and moderators of the treatment benefits that are found.

With respect to mindfulness meditation and NF in pediatric populations with chronic pain, there are too few clinical trials to be able to draw firm conclusions regarding efficacy. Preliminary data indicate that these treatments are well tolerated, and may be associated with improvements in some important pain outcome variables (e.g., pain intensity, frequency of pain episodes, well-being, physical function) in some populations. However, the long-term benefits of these treatments are not known. Critically, there is a lack of adequately designed randomized clinical trials evaluating the specific effects of these treatments. On the other hand, the side effect profiles of these treatments are positive; studies about meditation and NF do not report many (if any) significant negative effects associated to these treatments.

An additional important advantage of all of these psychological neuromodulatory treatments is that they promote and reinforce self-efficacy and encourage self-care. However, these advantages need to be weighed against the fact that these treatments require the involvement and motivation of the patient. Another disadvantage of NF treatment specifically is that it requires many sessions (as many as 40 or more) and special equipment, both of which increase the cost of this intervention.

We are far from having an adequate level of evidence to provide strong recommendations that hypnosis, NF, and training in meditation should be offered to all youth with chronic pain. However, the preliminary evidence reviewed here is promising, and supports the idea that additional studies about the efficacy of psychological neuromodulatory treatments are warranted. These future studies should include larger samples, use more robust methodologies (i.e., randomized controlled trials), and use manualized (i.e., standardized) interventions to develop an empirical foundation from which to make informed decisions about which treatment(s) should be offered to which patient(s). Ideally, these studies would be conducted in samples of children with a variety of chronic pain problems. For example, although we can conclude based on the available evidence that hypnotic treatment is efficacious for the management of chronic abdominal pain and has moderate evidence in relation to chronic headaches, studies in other chronic pain problems are needed.

Moreover, future research is needed to expand the criteria used to assess the efficacy of these treatments from a focus on pain-related domains (intensity, frequency or duration) to measures of function. The Initiative on Methods, Measurement, and Pain Assessment in Clinical Trials (PedIMMPACT; [78]) recommended a group of common outcome domains and measures for clinical trials, and it would be useful if all those future trials include as many as these domains as possible (i.e., pain intensity, global judgment of satisfaction with treatment, symptoms and adverse events, physical functioning, emotional functioning, role functioning, sleep, and economic factors).

As research in this area expands, and more is known regarding the efficacy and mechanisms of hypnosis, NF, and meditation training and practice in youths with chronic pain, we anticipate that it will provide the necessarily empirical basis for making treatment decisions and recommendations. This should increase the treatment options available to children, which will ultimately result in an improvement in the comfort and overall quality of life of these individuals.

## Figures and Tables

**Table 1:** Summary of reviewed studies.

Author(s) Country	Neuromodulatory Treatment/Study Design	Group and Intervention Description	Sample Description	Assessment Points and Outcome/Process Variables	Summary of Key Findings
Siniatchkin et al. [37] Germany	NF Non-randomized pilot study	*Groups* G1: NF treatment G2: Healthy controls G3: Waiting list *Session details* Number of sessions: 1 introductory session + 10 training sessions over 8 weeks. Session content: - 20 trials of baseline CNV recordings (reaction time paradigm), 10′ training/5′ break - 30 trials of increasing SCP negativity, 15′ training/5′ break - 30 trials of suppressing SCP negativity 15′ training/5′ break - 15 transfer trials of increasing SCP negativity 7′ training/3′ break - 15 transfer trials of suppressing SCP negativity 7′ training/3′ break	G1: *n* = 10 80% male Mean age = 10.5 years Dx.: Migraine without aura G2: *n* = 10 70% male Mean age = 11.6 years Dx.: Healthy children G3: *n* = 10 80% male Mean age = 9.9 years Dx.: Migraine without aura	*Assessment points* - Baseline - Post-treatment - 6-month follow-up *Outcome variables* - Average number of days with migraine - Duration of migraine episodes - Headache intensity - Accompanying symptoms (i.e., nausea/vomiting) - Medication intake - Amplitude of the SCPs	50% of the treatment group presented a 50% or greater reduction in the number of migraines a month after treatment. Migraine duration reductions were observed in treatment and waiting list groups. No significant changes in the waiting list group in accompanying symptoms (nausea, vomiting, intensity of migraine, or medication intake). No significant differences between the treatment group and the waiting list group in medication intake and migraine intensity. Successful suppression of SCPs’ amplitude in the treatment group.
Stokes & Lappin [38] USA	NF Case series	*One group only* NF + pirHEG + medication *Session details* Number of sessions: 40 sessions (30 NF + 10 pirHEG/thermal feedback) Session content: - 2 channels NF - Individualized protocols - 5 electrode placement: T3 I T4, C3 I C4, F3 I F4, FP1 I FP2, P3 I P4 - Auditory or visual feedback	*n* = 13 70% female Mean age = 13.4 years Age range = 9–21 years Dx.: Migraine	*Assessment points* - Baseline - Follow-up (variable: from 3 months to 2 years) *Outcome variable* - Average number of days with migraine	Significant decreases observed in the average number of migraine days from pre-treatment to follow-up.
Gauntlett-Gilbert et al. [53] United Kingdom	Meditation Case series	*One group only* ACT residential pain management program *Session details* Sessions duration: 90 h over 3 weeks Session content: - Three components: physical conditioning, activity management, and psychology - Psychology topics included: - Acceptance - Defusion - Present moment contact - Values - Committed action - Self-as-context	*n* = 98 75% female Mean age = 15.6 years Age range = 10.8–19.0 years Dx.: Idiopathic pain, complex regional pain syndrome, back pain, abdominal pain, pain associated with hypermobility	*Assessment points* - Pre-treatment - Post-treatment - 3-month follow-up *Outcome variables* - Physical disability - Social disability - Walk distance - Sit to stand - Pain intensity - Depression - Pain-specific anxiety - Perceived psychosocial development - Pain catastrophizing - Acceptance of pain - School attendance - Number of medications - Health care use	Significant pre- to post-treatment improvements observed in physical and social disability, walking distance, pain anxiety, pain catastrophizing, pain acceptance, school attendance and medication use that were maintained at follow-up. Significant pre- to post-treatment improvements in depression and perceived psychosocial development were observed, but these improvements were not maintained at follow-up. Significant pre- to follow up decrease in health care use. No significant differences in pain intensity at post-treatment and follow-up.
Hesse et al. [50] USA	Meditation Case series	*One group only* MBSR *Session details* Number of sessions: 8 weekly sessions Session duration: 2 h Session content: - Homework and incentives - Welcoming and centering practice: awareness and mindfulness sound - “Food for thought”: relations of quotes or poems with their experiences - Didactic lessons: awareness of breath, heartfulness, and body scan guided meditations - Learned mindful listening, eating, and walking - Discussion of home practice - Closing mindfulness practice - Journaling prompts - Home practice - Guided meditation once per day - Daily diaries	*n* = 20 100% female Mean age = 14.15 years Age range = 11–16 years Dx.: Headache	*Assessment points* - Pre-treatment - Post-treatment *Process variables* - Average number of sessions attended - Average of adherence to daily meditation - Completion rate - Helpfulness of the intervention - Perceived effect of the intervention to headache *Outcome variables* - Frequency and severity of headache - Pain interference - Headache disability - Quality of life (physical, emotional, social, and school function) - Pain acceptance: activity engagement and pain willingness - Depression - Anxiety	Average number of sessions attended: 6.10 of 8 total sessions. Average of adherence to daily meditation practice: 4.69 of 6 practices per week. Number (%) who completed treatment: 15 (75%). 53% reported the treatment was helpful in coping with stress, relaxing and controlling their emotions and pain; 40% reported that it was helpful in specific ways (i.e., pain reduction); 13% reported the intervention was not as helpful as expected. 33% reported the intervention not affect their headache, 20% reported having fewer headaches, 13.3% reported having less severe headache, and 7% the headache got better. Significant pre- to post-treatment improvements were observed in depressive symptoms and pain willingness. No significant pre- to post-treatment differences were observed in frequency and severity of headache, disability, quality of life, anxiety, or activity engagement.
Jastrowski et al. [51] USA	Meditation Randomized pilot (i.e., very small sample size) study	*Groups* G1: MBSR G2: Psycho-educational group *Session details* Number of sessions: 6 weekly sessions Session duration: 90’ Session content: G1: - Body awareness - Basic yoga - Relaxation techniques - Body-scan meditation - Walking meditation - Appreciation of the self and respect for uniqueness - Non-judgment of thoughts - Gratitude meditation - 30 min homework 6 days/week. G2: - Cognitive-behavioral model of chronic pain (anatomy-physiology and misconceptions about pain) - Stress management - Communications skills	G1: N = 4 75% female Mean age = 15.0 years Age range = 12–17 year. Dx.: Chest pain, extremity pain, headache pain, back pain G2: N = 2 100% female Mean age = 12.5 years Age range = 12–13 years Dx.: Abdominal pain	*Assessment points* - Pre-treatment - Post-treatment - 4-week follow-up - 12-week follow-up *Process variables* - Group attendance - Participants’ expectations about the benefits of MBSR - Helpfulness of the treatments components *Outcome variables* - Number of days with pain prior 2 weeks - Pain intensity - Pain duration - State and Trait Anxiety - Mindfulness self-efficacy - Quality of life (physical, emotional, social, and school functional domains) - Catastrophic thoughts - Functional disability	Average number of sessions attended: G1: 4/6 sessions G2: 3/6 sessions In general, participants had positive expectations of the proposed interventions. 75% of the participants in G1 reported expecting that MBSR would be “somewhat” to “completely helpful” at pre-treatment. At 12-week follow-up one participant reported that MBSR was “completely helpful” and another that it was “not at all helpful”. Qualitative individual analyses for outcome variables (data are missing for several participants and for different assessment points) indicate that: - Mindfulness self-efficacy increased for all participants in both groups. - Inconsistent results on the other outcomes measures.
Martin et al. [54] USA	Meditation Case series	*One group only* ACT *Session details* Number of sessions: 3 Session duration: 2 h over 2 days Session content: - Mindfulness techniques such as mindful breathing - Home practice: ACT exercises to practice between sessions	*n* = 10 50% girls Mean age = 16.9 years Age range = 12–20 years Dx.: Neurofibromatosis type 1	*Assessment points* - Baseline - 3-month follow-up *Process variables* - Treatment adherence - Satisfaction with treatment (adolescent and parents) *Outcomes variables* *Adolescents*: - Pain interference - Pain intensity - Functional disability - Pain acceptance - Pain-related anxiety - Depression - Health-related quality of life (daily, emotional and cognitive functioning, medical/physical status) - Pharmacological and non-pharmacological techniques used by to manage pain - Disease severity (completed by a nurse practitioner) *Parents* - Child pain interference - Acceptance of child’s pain - Health-related quality of life (daily, emotional and cognitive functioning, medical/physical status) - Psychological distress (e.g., anxiety, depression, and somatization)	60% of the participants used mindfulness techniques at least once a week at follow-up. Average participant satisfaction with study was 3.9 on a 0–5 scale. Average parent satisfaction with treatment was 4.6 on a 0–5 scale. Significant pre-treatment to follow-up improvements in pain intensity and pain interference were observed. 60% of the participants reported a decrease of medication at follow-up, relative to pre-treatment. Parents reported a significant pre-treatment to follow-up reduction in pain interference. No significant pre-treatment to follow-up improvements were reported in functional ability, anxiety, depression, quality of life by patient or parent reports, and acceptance of child’s pain.
Ruskin et al. [52] Canada	Meditation Pilot uncontrolled clinical study	*One group only* MBSR *Session details* Number sessions: 8 Session duration: 2 h Session content (meditation exercises): - Bringing comfort to pain - Kindness to pain - Body scan - Mindful eating - Breathing meditation - Mountain meditation - Loving kindness - Gratitude - Home practice: 5 min daily	*n* = 16 100% girls Mean age = 5.75 years Age range = 13–17 years Dx.: Neurophatic pain, musculoskeletal pain, abdominal pain, mixed pain, headache	*Assessment points* - Baseline - Post-treatment *Process variables* - Completion rate - Sessions attendance - Recommendation of treatment to others - Importance of learning and practice mindfulness - Confidence in using mindfulness - Helplessness of the intervention (i.e., to cope with pain, negative emotions and to be more kind with themselves) - Favorite activities of the treatment	Completion rate: 81% Average sessions attendance was 6.4 out of 8 sessions. All participants would recommend the intervention. Average importance of learning and practice mindfulness rated as 4.17 out of 5. Average confidence in use mindfulness rated as 4 out of 5. MBSR rated as being useful to cope with pain and negative emotions and for being more kind with themselves (average rating = 3.67 out of 5). Favorite activities of the treatment included: experiential exercises, meeting others with similar life experiences, group discussions, and learning new techniques to cope with pain. Areas of improvement noted: need of more specific and immediate techniques for managing pain flare-ups, need of more time to share pain stories with other participant, and difficulties with getting to the hospital after a school day.
Anbar &Zoughbi [64] USA	Hypnosis Case series	*One group only* Hypnosis *Session details* Number of sessions: Mean of 2 sessions of hypnosis in clinic with a mean of 3.8 sessions (range 1–16)	*n* = 30 56.6% female Mean age = 15 years Age range: 10–18 years Dx.: Headache	*Assessment points* - Baseline - Post-treatment - Follow-up (time not specified) *Outcome variables* - Headache frequency - Headache pain intensity	96% of the participants reported pre- to post-treatment decreases in headache frequency and intensity. Pre- to post-treatment improvements were maintained at follow-up for 65% of the sample.
Galini, Shaoul & Mogilner [62] Israel	Hypnosis Case series	*One group only* Hypnosis *Session details* Number of sessions: 1	*n* = 20 75% female Age range = 11–18 years Dx.: Chronic recurrent functional abdominal pain	*Assessment points* - Baseline - Post-treatment *Outcome variables* - Pain intensity - Pain frequency	70% of the participants reported pre- to post-treatment improvements in pain intensity and pain frequency.
Kohen & Zajac [63] USA	Hypnosis Case series	*One group only* Hypnosis *Session details* Number of sessions: 3 to 4	*n* = 144 66% female Mean age = 11.0 years Age range = 5–15 years Dx.: Headache	*Assessment points* - Baseline - Post-treatment *Outcome variables* - Headache frequency - Headache pain intensity - Headache duration	88% of the participants reported a decrease in headache frequency (from 4.5 to 1.4/week), 87% a decrease in headache pain intensity (10.3 to 4.7 in a 12-point scale), and 26% experienced a resolution in their headache. Headache duration decreased from 23.6 to 3.0 h, on average.
Olness et al. [70] USA	Hypnosis Randomized controlled trial	*Groups* G1: Placebo-placebo-hypnosis G2: Propranolol-placebo-hypnosis G3: Placebo-propranolol-hypnosis *Session details* Number of sessions: 3 during 12 weeks, 10-week placebo or drug treatment period.	G1: *n* = 9 44.4% female Mean age = 8.4 years Age range = 6–12 years Dx.: Migraine G2: *n* = 11 18.2% female Mean age = 9.6 years Age range = 6–12 years Dx.: Migraine G3: *n* = 8 62.5% female/male Mean age = 9.6 years Age range = 6–12 years Dx.: Migraine	*Assessment points* - Baseline - Post-treatment *Outcome variables* - Headache frequency - Headache pain intensity	Participants in the hypnosis group reported a significantly greater pre- to post-treatment decrease in headache frequency relative to control group, but no significant differences were found regarding pain intensity.
Van Tilburg et al. [66] USA	Hypnosis Randomized controlled trial	*Groups* G1: Standard medical care + listening to recorded hypnotic sessions G2: Standard medical care *Session details (G1)* Number of sessions: 3 biweekly sessions, including 1 booster session + 3 daily sessions. Treatment period: 2 months Session content: Listen to tape with self-exercises ≥5 days/week.	G1: *n* = 19 G2: *n* = 15 71% female Age range = 6–16 years Dx: Functional Abdominal Pain	*Assessment points* - Baseline - Post-treatment *Outcome variables* - Pain intensity - Composite score of quality of life (physical, emotional, social, and school functional domains) - School absenteeism - Medication use	Participants in the hypnosis group reported significantly greater pre- to post-treatment improvements in pain intensity and perceived “health related quality of life” than participants in the control group. No significant differences between the hypnosis and control groups were observed in school absenteeism or medication use.
Vlieger et al. [67] Vlieger et al. [68] * Netherlands	Hypnosis Randomized controlled trial	*Groups* G1: Hypnosis G2: Standard medical care + supportive therapy *Session details* Number of sessions: 6 Session duration: 50’over a 3-month period for the G1. Six 30’ session over a 3-month period for the G2.	G1: *n* = 27 67% female G2: *n* = 22 86% female Mean age = 13.2 years Age range = 8–18 years Dx.: Irritable bowel syndrome, functional abdominal pain	*Assessment points* - Pre-treatment - Post-treatment - 1-year follow-up - 5-year follow-up *Outcome variables* - Pain intensity - Pain frequency (days per month) - General improvement - School absenteeism	Participants in the hypnosis group reported significantly greater pre- to post-treatment improvements in pain intensity and frequency. Participants in the hypnosis group reported significantly greater general pain improvement at 1-year and 5-year follow up. No significant differences between the hypnosis and control groups were observed in school absenteeism at a 5-year follow-up.
Weydert et al. [69] USA	Hypnosis Randomized controlled trial	*Groups* G1: Standard medical care + 4 hypnosis sessions G2: Standard medical care + breathing techniques Session details Number of sessions: 4 weekly sessions Session content: G1: Progressive relaxation + guided imagery. Listen to tape with self-exercises twice a day. G2: Learning three breathing techniques.	G1: *n* = 14 77% female Mean age = 11.0 years Dx.: Abdominal pain G2: *n* = 8 50% female Mean age = 11.1 years Dx.: Abdominal pain	*Assessment points* - Pre-treatment - Post-treatment - 1-month follow up *Outcome variables* - Pain frequency - School absenteeism	Participants in the hypnosis group reported significantly greater pre- to post-treatment improvements in pain frequency that were maintained at 1-month follow up. Participants in the hypnosis group reported significantly greater pre- to post-treatment improvements in school absenteeism that were maintained at 1-month follow up.
Zeltzer et al. [65] USA	Hypnosis Case series	*One group only* Hypnosis *Session details* Number of sessions: 6 weekly sessions Session content: acupuncture combined with 20′ of hypnotic sessions.	*n* = 31 61% female Mean age = 13 years Age range = 6–18 years Dx.: Headache, abdominal pain associated with irritable bowel syndrome, fibromyalgia, complex regional pain syndrome, juvenile rheumatoid arthritis, myofascial back and chest pain	*Assessment points* - Pre-treatment - Post-treatment *Outcome variables* - Average pain intensity - Current pain intensity - Pain interference in functioning - Anxiety - Depression	Children: Participants reported significantly greater pre- to post-treatment improvements in current and average pain intensity; 42.5% of children reported a decrease in current pain. Participants reported significantly greater pre- to post-treatment improvements in pain interference in functioning. Participants reported significantly greater pre- to post-treatment improvements in anxiety (50% of children reported decrease). No significant changes were reported in depression. Parents: Parents reported significantly greater pre- to post-treatment improvements in current and average pain intensity as well as pain interference in functioning.

* This publication reports on the five-year follow-up of the sample. Data from there participants of the control group are missing; NF: Neurofeedback; SCP: Slow Cortical Potentials; pirHEG: passive infrared hemoencephalography; CNV: Contingent Negative Variation; ACT: Acceptance and Commitment Therapy; MBSR: Mindfulness-based stress reduction; T: temporal area; C: central area; F: frontal area; FP: prefrontal area; P: parietal area; G: group; *n*: number of participants; Dx.: Diagnosis.
